# Transition between social protection systems for workers with long term health problems: A controlled retrospective cohort study

**DOI:** 10.1016/j.ssmph.2023.101491

**Published:** 2023-08-14

**Authors:** Daniel Griffiths, Michael Di Donato, Tyler J. Lane, Shannon Gray, Ross Iles, Peter M. Smith, Janneke Berecki-Gisolf, Alex Collie

**Affiliations:** a School of Public Health and Preventive Medicine, Monash University, Melbourne Australia; b Institute of Work and Health, Toronto, Canada; c Accident Research Centre, Monash University, Melbourne, Australia

**Keywords:** Social security, Disability insurance, Legislative reform, Workers' compensation, Data linkage, Unemployment benefits

## Abstract

Many nations have established workers' compensation systems as a feature of their social protection system. These systems typically provide time-limited entitlements such as wage replacement benefits and funding for medical treatment. Entitlements may end for workers with long-term health conditions before they have returned to employment. We sought to determine the prevalence of transitions to alternative forms of social protection, specifically social security benefits, among injured workers with long-term disability, when workers' compensation benefits end. We linked Australian workers' compensation and social security data to examine receipt of social security payments one year before and after workers' compensation benefit cessation. Study groups included (1) injured workers whose workers' compensation benefits ceased due to reaching a 260-week limit introduced by legislative reform (N = 2761), (2) a control group of injured workers with at least 104 weeks workers compensation income support (N = 3890), and (3) a matched community control group (N = 10,114). Adjusted binary logistic regression examined the odds of transitions to social security in the injured worker groups relative to the community control group. Within 12 months of workers' compensation benefit cessation, 60% (N = 1669) of the exposed group received social security payments, of which 41% (N = 1120) received the unemployment allowance and 19% (N = 516) the disability pension. Among the work injured control group, 42% (N = 1676) received social security payments after workers compensation benefits ceased. Transitions to social security payments were significantly more common than community levels for both exposed (OR 25.0, 95%CI = 20.7, 30.1) and work injured control groups (OR 4.7, 95%CI = 4.2, 5.3). Many injured workers with long-term health problems transition to social security when their workers’ compensation benefits cease. Transitions were more common among workers whose claims ended due to legislative reform which time-limited benefits. Design and implementation of system level policy reform should consider the social and economic impacts of transitions between separate social protection systems.

## Highlights

• Few studies have examined transitions from workers' compensation to social security when workers' compensation benefits cease.
• This study linked state and national data to map injured worker transitions between in the state of New South Wales.
• 41% of injured workers with long-term health problems transitioned from workers' compensation to unemployment payments.
• A further 19% transitioned to the disability support pension when workers' compensation benefits ceased.
• These transitions were attributable to a policy reform in the New South Wales workers' compensation scheme.

## 1 Introduction

Globally, work-related injury and disease result in nearly 90 million disability-adjusted life years per annum (World Health Organization, 2021). For some workers, initial work-related health problems can lead to extended periods of time off work. During these periods, workers may access employment-injury benefits to replace lost income (Otting, 1993). Many countries have established workers’ compensation (WC) systems, which provide entitlements such as regular wage replacement benefits and funding for medical treatment. These entitlements usually end following a return to work, but entitlement periods for workers with long-term health conditions can also end upon reaching defined time limits, reaching retirement or age pension age, or upon settling a claim with a lump sum payment (Safe Work Australia, 2021). In some countries WC is an integrated part of a broader national social protection system (e.g. Brazil, France, Austria, Hungary, Sweden), whereas in other countries WC operates independently of social “safety net” systems at a state or provincial level (e.g. Australia, Canada, USA). For the latter group, workers may transition from WC to a separate social security (SS) system if they remain unable to work when WC entitlements end.

Despite these transitions being a feature of social protection in many nations, there have been very few studies examining transitions from WC to SS payments (e.g. for unemployment or disability). A national cross-sectional study in Australia identified that people receiving SS payments after WC benefits ceased also reported high levels of financial stress (Sheehan et al., 2020). In the USA, many people with low back pain WC claims transitioned to SS disability insurance (Chibnall et al., 2006), while workers with persistent health conditions had increased likelihood of receiving disability payments in the state of New Mexico (O'Leary et al., 2012). Also in the USA, changes to WC systems that reduced benefit generosity were associated with a significant increase in SS disability insurance applications (Guo & Burton, 2012). In the province of British Columbia, Canada, a data linkage study reported that 24% of workers injured in 1991 received SS income support payments between 1990 and 1997, compared to less than 10% of a matched community cohort (Hertzman et al., 1999). Much of the aforementioned evidence is from the 20th century, and describes social protection systems which have since undergone substantial reform. Updating the evidence base with more recent data, and directly measuring the movement of individual workers between social protection systems, will be beneficial for understanding current approaches, and informing future policy to best support people with long-term work disability.

Reform of government social policies represent potentially valuable natural experiments, particularly where population level data is available to evaluate the impact of such reform (Collie et al., 2020). This study exploits a major reform to the WC legislation in the Australian state of New South Wales (NSW) which came into effect on October 1, 2012. The reform affected entitlements to benefits for several thousand workers with long duration compensation claims. Like other Australian states, NSW operates a no-fault, compulsory, cause-based WC scheme that provides financial support and funded healthcare to workers with an injury or illness that can be attributed to their employment (Safe Work Australia, 2021; Collie et al., 2019). Workers with injury resulting in permanent impairment may also be eligible for a lump-sum payment. Prior to the 2012 reforms injured workers with accepted claims could receive WC entitlements until they reached 65 years of age, the age of eligibility for the Australian aged pension. Under the legislative reform WC entitlements were capped at 260 weeks (i.e. 5 years) from 2012, with the first cohort workers receiving their final income support benefit from late 2017 (State Government of New South Wales, 2012). These workers remain eligible for funded medical treatment for up to two years beyond the end of their income support benefits.

This study examines receipt of SS payments of this cohort after their WC income support benefits end. For injured workers, the most relevant SS income support payments in Australia include the unemployment payment and the Disability Support Pension (DSP). Eligibility criteria for each payment depend on personal and situational factors such as age, income and assets, relationship status, compliance with mutual obligations such as job seeking behaviour, and residency rules. The DSP typically provides higher payments than the unemployment payment, but also requires applicants to meet additional medical and functional eligibility criteria. Eligibility for these payments differ greatly from those in the WC systems (Collie et al., 2019). This study sought to measure the prevalence of transitions from WC benefits to different types of SS payments in workers with long-term health conditions affecting their work ability.

## 2 Methods

### 2.1 Setting

The Australian state of NSW had a resident population of 7.3 million people in 2012 (Australian Bureau of Statistics, 2023), with a labour force of 3.83 million (Australian Bureau of Statistics, 2012, p. 6291) In 2022 the Australian Government Department of Social Services reported that 258,523 people in NSW received the unemployment payment, 239,252 received the DSP, 791,951 received the aged pension, and 109,583 received the carer payment (for providing constant care to someone who has a severe disability or illness) (Department of Social Services, 2022).

### 2.2 Workers’ compensation reform

In NSW about 100,000 W C claims are made each year (State Insurance Regulatory Authority, 2023). In 2012, a major reform of the state WC scheme restricted eligibility for most workers, introduced time limits on the duration of income support, and capped durations of future medical care coverage in response. The reforms were in response to a deteriorating financial position, which included projections of an unfunded liability of AUD$4.1 billion and a 28% increase in employer's insurance premiums (Centre for International Economics, 2014). One major element of the reforms included that income support benefits were capped at 260 cumulative weeks (equivalent to 5 years) maximum duration, with exemptions made for people with whole person impairment assessed as being greater than 20%. These reforms were described in Section 39 of the state Workers' Compensation Legislation Amendment Act 2012; State Government of New South Wales, 2012), which came into effect on October 1, 2012 The change was prospective, meaning that benefits began to cease under Section 39 from late 2017, five years after the reforms were introduced.

### 2.3 Study design

This is a controlled retrospective cohort study using linked WC and SS data. The study protocol has been previously published (Lane et al., 2021) describing the linkage of multiple databases at a case/person level including records of NSW WC claims, with (1) NSW hospital admission and emergency department data, (2) health care data funded by the Australian Medicare scheme; and most importantly for this study (3) data extracted from the Australian Government Department of Social Services Data Over Multiple Individual Occurrences (DOMINO) database, which includes social security income support payments.

### 2.4 Study groups

#### Three study groups were defined

**1. Section 39 group**: The first cohort of injured-workers whose WC income benefits stopped from late 2017 to mid 2018 due to the legislative reform,

**2. Work Injured Control group**: Injured workers who had received at least 104 weeks of WC income benefits and whose benefits ended, but not due to the legislative reform,

**3. Community Control group**: A group of people drawn from the general population and matched to Section 39 group on age, sex and residential geographic location.

The Section 39 and Work Injured Control groups had incurred a work-related injury, illness or disease and had an accepted WC claim. The Section 39 group been exposed to WC for at least five years. The Work Injured Control group had been exposed to WC for at least 2 years. Members of the Community Control group had not been exposed to WC, other than a small percentage expected at a population level for people with the corresponding demographic profile. In addition, Section 39 group was exposed to the enforced cessation of income benefits by legislative reform, whereas the Work Injured Control group was not.

### 2.5 Study periods

Each person was assigned an index date. The study period for each person consisted of the 12-month interval prior to the index date (i.e. ‘before’), and the following 12-month interval (i.e. ‘after’). For members of Section 39 and Work Injured Control groups, index dates were set as the date of the final WC income benefit. For the Community Control group, index dates were set as December 25, 2017, which was the median index date of Section 39 group.

### 2.6 Eligibility criteria

**Fig. 1:**
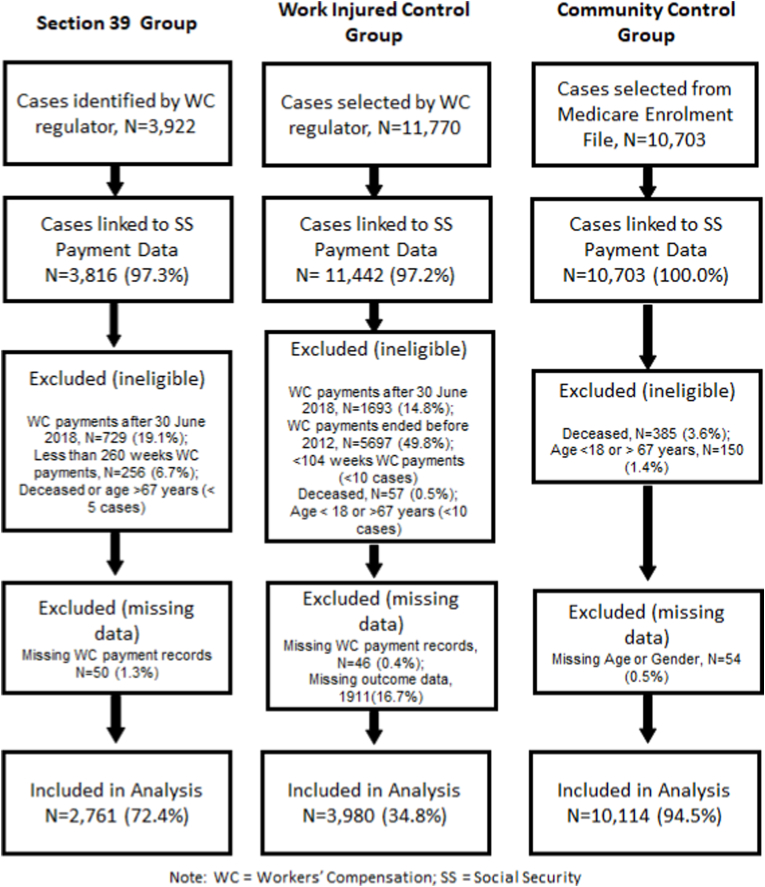
Flowchart describing linkage rates and application of study eligibility criteria to linked cases.

During 2017, insurers for the NSW WC scheme identified potential future members of Section 39 group. People within this group who did not attain 260 weeks of workers’ compensation income benefits (by June 30, 2018) were excluded from Section 39 group (Fig. 1). Within the Work Injured Control group, people that did not attain 104 weeks of WC income benefits (by June 30, 2018) were excluded. Index dates were required to be within 26 Sept 2017–June 30, 2018 for Section 39 group corresponding to the earliest benefit cessation date under Section 39 and to allow a 12 month follow-up period to measure study outcomes. Index dates were required to be within Jan 1, 2012–June 30, 2018 for the Work Injured Control group corresponding to a post-2012 reform period of the WC system. People aged 18–67 years at their index date were included in the study, and they were required to be aged at least 18 years at the beginning of their WC claim. Individuals who died more than two weeks prior to the index date were excluded.

### 2.7 Study outcomes

Study outcomes describe receipt of a SS income support payment across five binary outcome categories based on the type of payment received (Supplementary Table 1 for more detail): (1) Any income support payment, (2) the unemployment payment, (3) the disability payment, the (4) age pension, and (5) the carer payment.

Payments within two weeks of an index date were excluded from analyses to increase confidence of assigning outcomes to the most relevant study period. Outcomes are reported as counts and study group percentages for each study period.

### 2.8 Analytical approach

Generalised estimating equations (GEEs) assessed the relationship between study groups and outcomes 1 to 3. Statistical models did not examine receipt of the Age Pension (i.e. outcome 4) because this payment is exclusively for those aged above normal retirement age, nor receipt of the Carer payment (i.e. outcome 5) due to relatively small sample sizes. Exposure groups within each regression model describe six pairwise interaction terms of three study groups with two study periods. Post-hoc tests evaluated pairwise differences between study groups during the post-index study period, whilst controlling for differences during the pre-index study period, by computing a linear combination of coefficients from the regression model, using the STATA Rlincom package Covariates corresponding with eligibility criteria for SS payments were chosen from those available, including gender, age group, marital/relationship status, having children aged under 18 years, home ownership, country of birth, socioeconomic area of residence, and remoteness. Socio-economic status and remoteness were based on postcode of primary residence. All covariates with the exception of country of birth were as reported at the index date. Modelling was repeated for each outcome with an additional set of covariates derived from compensation data including the nature and location of compensable injury, the duration of income from WC, and whether the person had been involved in any legal action during their WC claim. The additional set of regression models did not include the Community Control group as this group did not have data available from compensation records. Unadjusted and Adjusted Odds Ratios (AORs) with 95% Confidence Intervals (CIs) were estimated from GEE models. Cases with missing data were excluded from analysis.

## 3 Results

### 3.1 Data linkage and eligibility

From 15,692 WC records, 15,258 (97%) were linked to the SS payment dataset. Application of eligibility criteria resulted in the exclusion of 8517 linked cases (56%), of which 5697 (36%) had index dates prior to 2012 (i.e. only part of the pre-reform WC system), and 2426 (16%) received compensation after June 30, 2018. Following application of eligibility criteria linked cases reduced from 3816 to 2761 (72%) for Section 39 group, 11,442 to 3980 (35%) for the Work Injured Control group, and 10,703 to 10,114 (94%) for the Community Control group (Fig. 1).

### 3.2 Group characteristics

Within Section 39 group, 41% had received WC income benefits for between 5 and 10 years, and 59% received income benefits for more than 10 years. Within the Work Injured Control group, 46% had received WC income benefits for 2–5 years, 29% for 5–10 years and 25% for more than 10 years. Age distributions differed between study groups, and Section 39 group and Community Control group had a higher percentage of women (46% and 45% respectively) than the Work Injured Control group (36%). The most common location of injury was the central body (i.e. trunk) which was more common in Section 39 group (43%) compared to the Work Injured Control group (35%). A more detailed profile of each study group is provided in Table 1.

**Table 1:** Characteristics of the study groups.

Study group	Section 39	Work Injured control	Community control
	N (%)	N (%)	N (%)
**Gender**			
Female	1269 (46.0)	1508 (37.9)	4578 (45.3)
Male	1492 (54.0)	2472 (62.1)	5536 (54.7)
**Age**			
18–34 years	45 (1.6)	282 (7.1)	330 (3.3)
35–44 years	218 (7.9)	653 (16.4)	1109 (11.0)
45–54 years	750 (27.2)	1184 (29.7)	2950 (29.2)
55–64 years	1508 (54.6)	1200 (30.2)	5289 (52.3)
65 or more years	240 (8.7)	661 (16.6)	436 (4.3)
**Relationships/Marital status**			
Single without dependent children	946 (34.3)	1207 (30.3)	2882 (28.5)
Partnered without dependent children	1147 (41.5)	1595 (40.1)	3373 (33.3)
Single with dependent children	272 (9.9)	423 (10.6)	894 (8.8)
Partnered with dependent children	341 (12.4)	641 (16.1)	1751 (17.3)
Unknown	55 (2.0)	114 (2.9)	1214 (12.0)
**Homeownership**			
Yes	1398 (50.6)	1895 (47.6)	4658 (46.1)
No	1076 (39.0)	1590 (39.9)	3936 (38.9)
Unknown	287 (10.4)	495 (12.4)	1520 (15.0)
**Country of birth**			
Australia	1807 (65.4)	2702 (67.9)	6999 (69.2)
Not Australia	925 (33.5)	1213 (30.5)	2909 (28.8)
Unknown	29 (1.1)	65 (1.6)	206 (2.0)
**Socioeconomic area**			
Moderate	1379 (58.1)	2033 (51.1)	4298 (56.0)
Advantaged	192 (8.1)	365 (9.2)	749 (9.8)
Disadvantaged	803 (33.8)	1094 (27.5)	2624 (34.2)
Unknown	387 (14.0)	488 (12.3)	2443 (24.2)
**Remoteness**			
Major Cities	1521 (55.1)	2408 (60.5)	4961 (64.7)
Regional	841 (30.5)	1074 (27.0)	2667 (34.8)
Remote	12 (0.4)	12 (0.3)	44 (0.6)
Unknown	387 (14.0)	486 (12.2)	2442 (24.1)
**Nature of injury**			
Injury and poisoning	2225 (80.6)	3131 (78.7)	–
Musculoskeletal System and Connective Tissue	245 (8.9)	349 (8.8)	–
Mental disorders	187 (6.8)	375 (9.4)	–
Other	61 (2.2)	97 (2.4)	–
Unknown	43 (1.6)	28 (0.7)	–
**Location of injury**			
Head	45 (1.6)	69 (1.7)	–
Lower Limbs	361 (13.1)	556 (14.0)	–
Multiple locations	287 (10.4)	461 (11.6)	–
Neck	56 (2.0)	103 (2.6)	–
Non-physical locations	187 (6.4)	383 (9.6)	–
Systemic locations	8 (0.3)	12 (0.3)	–
Trunk	1179 (42.7)	1376 (34.6)	–
Unknown	41 (1.5)	24 (0.6)	–
Upper limbs	597 (21.6)	996 (25.0)	–
**Weeks of Entitlement**			
104–259 weeks (2–5 years)	–	1849 (46.5)	–
260–311 weeks (5–6 years)	311 (11.3)	341 (8.6)	–
312–519 weeks (6–10 years)	824 (29.8)	810 (20.4)	–
520–779 weeks (10–15 years)	770 (27.9)	721 (18.1)	–
780 weeks or more (15+ years)	856 (31.0)	259 (6.5)	–
**Common law action**			
Yes	170 (6.2)	507 (3473)	–
None recorded	2591 (93.8)	3473 (87.3)	–
**Total**	*2761 (100.0)*	*3980 (100.0)*	*10,114 (100.0)*

Notes: Time-dependent values are described at the workers' compensation time loss payment cessation date, which is defined as Dec 25, 2017 (i.e. median cessation date of Section 39 group) for the community control group.

### 3.3 Transitions to any social security payments

During the post-index study period, 60% of Section 39 group received SS payments compared to 7% in pre-index study period. In the Work Injured Control group, 42% received SS payments in the post-index period compared to 14% in the pre-index period. Receipt of SS payments in the Community Control group increased from 27% to 30% between the study periods (Table 2). After controlling for differences in receipt of SS in the pre-index period, the adjusted odds of receiving any SS payment in the post-index period was 25 times higher (AOR 25.0, 95% CI = 20.7, 30.1) for Section 39 group and 5 times higher (AOR 4.7, 95% CI = 4.2, 5.3) for the Work Injured Control group, compared to the Community Control group (Table 3). After additionally accounting for differences in compensable injury claims, the odds of receipt of SS payments by Section 39 group in the post-index period was 5 times higher (AOR 5.3, 95% CI = 4.3, 6.5) than the Work Injured Control group.

**Table 2:** Number (percentage) of people in study groups receiving social security payments in the year before or after workers' compensation benefit cessation.

Outcome (Type of Payment)	Section 39 Group		Work Injured Control Group		Community Control Group	
	Before	After	Before	After	Before	After
Any income support payment	191 (6.9)	1669 (60.4)	559 (14.0)	1676 (42.1)	2689 (26.6)	3024 (29.9)
Unemployment payment	28 (1.0)	1120 (40.6)	197 (4.9)	623 (15.7)	951 (9.4)	914 (9.0)
Disability support pension	139 (5.0)	516 (18.7)	176 (4.4)	336 (8.4)	1148 (11.4)	1172 (11.6)
Aged pension	11 (0.4)	209 (7.6)	144 (3.6)	516 (13.0)	69 (0.7)	303 (3.0)
Carer payment	14 (0.5)	110 (4.0)	30 (0.8)	91 (2.3)	403 (4.0)	402 (4.0)
No payment	2570 (93.1)	1092 (39.6)	3421 (86.0)	2304 (57.9)	7425 (73.4)	7090 (70.1)
**Group Total**	***2761 (100.0)***		***3980 (100.0)***		***10,114 (100.0)***	

Note: All data are presented as frequency (percentage of group).

**Table 3:** Generalised estimating equations for social security payments received during the 12 months before or after workers’ compensation benefit cessation.

Model A. Unadjusted Odds Ratios [95% CIs]	Any social security payment	Disability Support Pension	Unemployment Payment
Exposure: Timing of payment * Study group			
Before * Section 39	0.24* [0.22, 0.26]	0.56* [0.50, 0.62]	0.15* [0.14, 0.17]
Before * Work Injured control	0.50* [0.46, 0.54]	1.39* [1.22, 1.58]	0.56* [0.50, 0.63]
Before * Community control	0.85* [0.83, 0.87]	0.98* [0.96, 0.99]	1.05 [1.00, 1.10]
After * Section 39	4.87* [4.18, 5.68]	2.42* [2.02, 2.90]	10.15* [6.96, 14.82]
After * Work Injured control	2.22* [2.01, 2.45]	2.77* [2.35, 3.26]	2.00* [1.71, 2.34]
After * Community control	1.00 (ref.)	1.00 (ref.)	1.00 (ref.)
**Post hoc-tests**			
Difference between Section 39 and Community after (controlling for differences before)	17.46* [14.98, 20.35]	4.24* [3.62, 4.95]	69.52* [47.71, 101.30]
Difference between Work Injured group and Community after (controlling for differences before)	3.78* [3.45, 4.14]	1.95* [1.70, 2.23]	3.72* [3.20, 4.32]
Difference between Section 39 group and Work Injured group after (controlling for differences before)	4.62* [3.88, 5.50]	2.18* [1.77, 2.67]	18.69* [12.54, 27.87]
**Model B. Adjusted Odds Ratios [95% CIs]**	**Any social security payment**	**Disability Support Pension**	**Unemployment Payment**
Exposure: Timing of payment * Study group			
Before * Section 39	0.26* [0.23, 0.29]	0.64* [0.56, 0.74]	0.15* [0.13, 0.17]
Before * Work Injured Control	0.58* [0.53, 0.64]	1.21* [1.04, 1.41]	0.54* [0.47, 0.61]
Before * Community Control	0.82* [0.79, 0.85]	0.98* [0.96, 1.00]	1.05 [1.00, 1.11]
After * Section 39	7.81* [6.52, 9.36]	2.71* [2.24, 3.28]	11.25* [7.71, 16.42]
After * Work Injured Control	3.32* [2.93, 3.76]	2.37* [1.98, 2.83]	2.07* [1.74, 2.47]
After * Community Control	1.00 (ref.)	1.00 (ref.)	1.00 (ref.)
**Post hoc-tests**			
Difference between Section 39 and Community after (controlling for differences before)	24.95* [20.66, 30.13]	4.11* [3.49, 4.83]	80.22* [54.77, 117.50]
Difference between Injured group and Community after (controlling for differences before)	4.68* [4.15, 5.27]	1.91* [1.64, 2.21]	4.05* [3.41, 4.80]
Difference between Section 39 group and Work Injured group after (controlling for differences before)	5.34* [4.30, 6.62]	2.16* [1.73, 2.68]	19.83* [13.16, 29.88]
**Model C. Adjusted Odds Ratios [95% CIs]**	**Any social security payment**	**Disability Support Pension**	**Unemployment Payment**
Exposure: Timing of payment * Study group			
Before * Section 39	0.08* [0.07, 0.09]	0.27* [0.22, 0.33]	0.07* [0.06, 0.09]
Before * Work Injured Control	0.18* [0.16, 0.20]	0.51* [0.44, 0.59]	0.26* [0.22, 0.30]
After * Section 39	2.31* [1.89, 2.83]	1.16 [0.90, 1.50]	6.02* [3.95, 9.19]
After * Work Injured Control	1.00 (ref.)	1.00 (ref.)	1.00 (ref.)
**Post hoc-tests**			
Difference between Section 39 group and Work Injured control after (controlling for differences before)	5.27* [4.26, 6.51]	2.21* [1.77, 2.76]	21.06* [13.75, 32.27]

**Notes: Model A** is not adjusted for demographics or details of injury claim. **Model B** is the same as Model A but also adjusted for whether a corresponding social security payment was received within 12 months prior to workers' compensation cessation, gender, age group, partner and dependent children status, homeownership, country of birth, socioeconomic area, remoteness. Model B was not adjusted by details describing injury or workers' compensation claims.**Model C** is the same as Model B and additionally adjusted for data on the injury and compensation claim factors: Weeks of Entitlement, Nature of Injury, Location of Injury, Common law action.

### 3.4 Transitions to the unemployment payment

Unemployment was the main type of payment received during the post-index study period. In total, 41% of Section 39 group and 17% of the Work Injured Control group received unemployment payments, compared to 9% of the community control group (Table 2). After controlling for differences in receipt of unemployment payments in the pre-index period, the adjusted odds of receiving the unemployment payment within the post-index study period was 80 times higher (AOR 80.2, 95% CI = 54.8, 117.5) for Section 39 group and 4 times higher (AOR 4.1, 95% CI = 3.4, 4.8) for the Work Injured Control group, compared to the Community group. After also adjusting for differences in compensable injury claims, the odds were 20 times higher (AOR 19.8, 95% CI = 13.2, 29.9) for Section 39 group compared to the Work Injured Control group (Table 3).

### 3.5 Transitions to the disability support pension

The DSP was received by 19% of Section 39 group, 8% of the Work Injured Control group, and 12% of the Community Control group in the post-index period (Table 2). The odds of receiving the DSP in the post-index period was four times higher (AOR 4.1, 95% CI = 3.5, 4.8) for Section 39 group and nearly two times higher (AOR 1.9, 95% CI = 1.6, 2.2) for the Work Injured Control group, compared to the Community Control group. After also accounting for differences in compensable injury claims, the odds of receiving the DSP in the post-index period for Section 39 group were two times higher (AOR 2.2, 95% CI = 1.8, 2.8) than for the Work Injured Control group (Table 3).

### 3.6 Other income support payments

Receipt of other forms of working-age SS income support payments was relatively uncommon, with the carer payment being the next most prevalent payment increasing from 1% to 4% for Section 39 group over the study periods, 1%–2% for the Work Injured Control group, and remaining stable at 4% across study periods for the Community Control group (Table 2). Post-index, the aged pension was most common for the Work Injured Control group (13%), followed by Section 39 group (8%) and the Community Control group (3%).

## 4 Discussion

Our findings demonstrate that transitions to SS payments are common after WC income benefits end for workers with long-duration compensation claims. Among those whose WC benefits ceased due to the introduction of a legislated 260-week limit, most (60%) received SS payments in the following year. About two-thirds of those who transitioned to SS payments received unemployment payments, and a third received disability payments. The work injured control group, who were not exposed to the legislative reform, had lower rates of transition to SS payments, however these remained significantly higher than those observed in a community control group. To our knowledge this study is the first to examine, using linked case-level data, transitions to SS payments after WC benefits cease. The international studies in this area have generated mostly indirect evidence, covering broad time periods (O'Leary et al., 2012), or evaluating macro trends of disability benefit receipt following changes to compensation system rules (Guo & Burton, 2012).

Our study findings imply that many injured workers with long periods of work disability do not return to work following the end of their WC benefits. We demonstrate a surge of transitions to the nationally operated social ‘safety-net’ system, triggered by legislative reform in a separate state-based WC system. One consequence of the NSW legislative reform is that many of the long-term injured workers will have moved to much lower fortnightly payments from the SS system than they were receiving in the WC system. This is because WC payments are calculated as a percentage of pre-injury earnings, whereas SS payments represent a flat “safety net” rate that is typically much lower (Collie et al., 2019). The basic rate for the Australian unemployment payment in mid-2018 (during the follow-up period of this study) was $275.50 per week. Prior studies show that Australian SS recipients are likely to experience financial hardship and associated health impacts in addition to navigating stressful administrative processes and job-seeking/training obligations to receive benefits (Davidson et al., 2020; Milner et al., 2020; Sheehan et al., 2020). Dedicated supports such as financial advice, education on SS system processes or administrative support to apply for SS benefits, may act to alleviate some of these consequences of, and may support more timely access to benefits (Iles, 2022).

Overall, our findings are consistent with previously reported uptakes in disability payments for people with long-duration workers compensation claims (O'Leary et al., 2012; Guo & Burton, 2012), albeit under different circumstances, and we furthermore offer the first insights into the different forms of SS received across a diverse set of payment types. Transitions between types of income support payments within the Australian SS system itself have been well described (Department of Social Services, 2016), whereas our data linkage approach adds value by observing transitions between independent systems within the overall social protection framework. Our findings are most relevant to nations where compensation for work-related injuries and occupational diseases operate independently of national social welfare systems.

We also observe that 40% of workers with very long duration compensation claims did not transition to the Australian SS system when their WC benefits ended. It is possible but unlikely that many of these people returned to work upon the cessation of their WC benefits. It is more likely that they did not meet the eligibility criteria for SS payments, which are different to those in WC systems. For instance, in Australia, eligibility for unemployment and disability payments is based on means- and asset-testing criteria that consider spousal income and homeownership status for example, which are not reflected in the rules for accessing workers' compensation income benefits. Closer inspection of our data supports this hypothesis. For example, 74.7% of single (non-partnered) people in Section 39 group received a SS payment in the period after WC benefit cessation, compared with 46.8% of partnered people without dependent children. Further, 49.5% of people in Section 39 group receiving a SS payment after WC benefit cessation owned their own home, compared with 68.9% of people who did not receive a SS payment. Welfare stigma may represent an additional barrier to accessing SS unemployment payments for some eligible individuals (Baumberg, 2016).

Many people with long durations of work disability exit workers' compensation schemes every year without returning to work. Individuals' circumstances will differ, as will the nature of their work disability and prospects for alternative sources of income or delayed return to work. Further research is needed to monitor the welfare and health outcomes for these workers into the future to support the economic and human costs of employment-related health problems. Transitions from workers’ compensation to social security not only results in changes to income benefits, but also to healthcare coverage. Whilst this was not the purpose of this study, future research should also examine changes in the delivery of healthcare and access to medical benefits, and the impact any changes have on the health of people with work disability.

Aside from work income and social security payments, there are a myriad of financial supports systems available within Australia which have not been explored in this study, the most common of which may be a compulsory form of employer-contributed retirement pension (superannuation), and clauses associated with withdrawal of payments upon eligible circumstances including forms of temporary or permanent disability. Prior studies have shown that some workers rely on other source of income such as from their partners or other family members (Sheehan et al., 2020). It is also plausible that some people transition to SS payments more than one year after their workers’ compensation income benefits ceased. These transitions could not be observed in this study. This is more likely to occur for the disability payments it consists of a multi-stage application process, which for many applicants requires compiling original medical evidence and other supporting documentation, attending medical and job capacity assessments, and completing an 18-month period of job-searching/training (Collie et al., 2022).

Our findings are limited by potential for uncontrolled confounding. For example, whilst all injured workers included in the study were in paid work prior to their WC claim, this criteria could not be applied to the community control group. Furthermore, the community control group may have contained people in occupations which were excluded from Section 39 group such as firefighters and police officers. Other limitations include the lack of data on people who do not transition to SS payments from WC benefits. These groups represent a large proportion of both Section 39 and Work Injured Control groups, yet we are blind to the sources of financial support (if any) they access in the year after workers’ compensation benefit cessation. We have insufficient data to conduct analysis on the precise level of financial support provided to people in the WC and SS systems. While it seems clear that SS payments are capped at a much lower level than those available through WC, we cannot present data on the actual amount of payments made.

Study strengths include the large sample sizes, enabling us to estimate with confidence the proportion of injured worker groups transitioning from WC to SS benefits. Section 39 group included complete capture of those exposed to Section 39 legislative amendment during its period of implementation from late 2017 until mid-2018, limiting selection bias. Another strength is the inclusion of a matched community control group, which enabled comparison of transitions to SS payments among the injured groups to that occurring in the community. The data linkage method achieved very high rates of linkage, providing confidence in the representativeness of our findings. Linking retrospective data enabled us to examine SS receipt both before and after cessation of WC benefits, and to incorporate the ‘before’ state into statistical analysis, providing a more accurate estimate of the rate of transition once WC benefits ceased. Social security data was sufficiently detailed to enable examination of different types of financial payments providing a more nuanced understanding of transitions between systems.

## 5 Conclusion

The introduction of a 260-week duration limit on benefits in the state of New South Wales WC system led to many people transitioning to SS payments such as unemployment or disability benefits. Design and implementation of system-level policy changes within any single social protection system should consider both the potential health, economic and social impacts on individuals, but also the flow-on impacts to other social protection systems. Provision of targeted support upon cessation of WC benefits for people with continued impaired work capacity may ease the transitions to alternative income support systems such as social security, and help to mitigate any negative impacts associated with reduced financial resources, administrative burden and long-term unemployment.

## Declaration of interest

The authors declare that they have no competing interests.

## Competing interests

The authors declare that they have no competing interests.

## Funding

This project was funded by the State Insurance Regulatory Authority of New South Wales through a grant to the corresponding author (AC) via his institution. AC was funded by an Australian Research Council Future Fellowship (FT190100218).

## Authors' contributions

AC conceived the study. TL and AC managed the data linkage process. All authors prepared the study protocol. DG conducted analysis, which was reviewed by all authors. DG and AC drafted the manuscript. All authors reviewed the data analysis, contributed to drafting the manuscript, read and approved the final manuscript.

## Data provision

This publication uses data supplied by the State Insurance Regulatory Authority of New South Wales, the New South Wales Government and the Australian Government. The views expressed are the authors and are not necessarily the views of data providers.

## Availability of data and material

Restrictions apply to the availability of the study data, which were used under license for the current study, and so are not publicly available.

## Ethics approval

This study received ethics approval from the Monash University Human Research Ethics Committee on 21st Sept 2018 (Project 14,696), the New South Wales population and health services research ethics committee on June 28, 2019 (2019/ETH00422), and from the Australian Institute of Health and Welfare ethics committee on September 25, 2018 (EO 2018/4/480).

## Data Availability

The authors do not have permission to share data.
