# Supplementary material for: Transition between social protection systems for workers with long term health problems: A controlled retrospective cohort study
Source: SSM Popul Health. 2023 Aug 14;23:101491. doi: 10.1016/j.ssmph.2023.101491 (PMC10462876; doi:10.1016/j.ssmph.2023.101491)
Supplement: Multimedia component 1 [file mmc1.docx]

**SUPPLEMENTARY MATERIAL**

Supplementary Table 1. Overview of income support from the New South Wales workers’ compensation system and the major Australian social security basic payments.

| **Workers’ compensation weekly benefits in New South Wales** | |
| --- | --- |
| Wage replacement benefits | Income for loss of work (i.e. time loss counted in *Weeks of Entitlement*) for people who were injured in the workplace or during the course of employment. Injured workers in receipt of this payment have an accepted claim for work loss due to injury, illness or disability. These payments are subject to the 1987 Workers’ Compensation Act NSW and the cap on weeks of entitlement introduced by Section 39 at 260 weeks (i.e. 5 years). |
| **Social security income support payments included in this study** | |
| Any income support payment | Any of the available social security payments including the unemployment payment, the DSP, the aged pension, the carer payment and a range of other payments avialable in the Australian social security system including the parenting payment, sole parent pension, youth allowance, sickness allowance, special benefit, farm household allowance, Ausstudy or Abstudy. |
| Unemployment payments | Known as the Jobseeker Allowance, this is the main working age income support payment for people who have the capacity to work now or in the future. Eligibility includes being aged 22 years to Age Pension age, unemployed or temporarily unable to work or study due to illness or injury. |
| Disability support pension (DSP) | The DSP is an income support payment for people who are unable to support themselves through employment due to a permanent physical, intellectual or psychiatric impairment. Eligibility includes having a permanent physical, intellectual or psychiatric impairment assessed at 20 points or more under the Impairment Tables, and be unable to work or be retrained for work of at least 15 hours per week within the next two years due to impairment. |
| Aged Pension | The main income support payment for people who have reached Age Pension age. The Age Pension Age was 65 years and 6 months from 1 July 2017 to 30 June 2018, and 66 years from 1 July 2019 to 30 June 2021. |
| Carer Payment | Carer Payment is an income support payment for people who provide constant care for a person with a disability or severe medical condition. |
